# Temporal trends and developmental trajectories of physical fitness among university students: a 13-year accelerated longitudinal study

**DOI:** 10.3389/fpubh.2026.1896880

**Published:** 2026-08-19

**Authors:** Jia-Yu Lu, Yan-Ping Zhao, Jia-Yi Wu, Dan-Ni Wang, Kai-Hua Chen

**Affiliations:** 1 Department of Health Promotion and Behavioral Sciences, School of Public Health, Anhui Medical University, Hefei, Anhui, China; 2 School of Physical Education and Health, Anhui Medical University, Hefei, China

**Keywords:** accelerated longitudinal design, cohort effects, longitudinal trajectories, physical fitness, university students

## Abstract

**Background:**

University represents a critical developmental stage during which physical fitness may undergo substantial changes. However, evidence regarding long-term physical fitness trajectories and cohort differences among university students remains limited, particularly in the context of the COVID-19 pandemic.

**Methods:**

This study used annual physical fitness surveillance data collected from 2013 to 2025. Partial follow-up data from the 2012 enrollment cohort were additionally included to reconstruct developmental trajectories across university years using an accelerated longitudinal design. A total of 50,158 students contributing 143,292 observations were included. Interrupted time-series analyses were conducted to assess temporal changes in physical fitness before and after 2020. Linear mixed-effects models were used to examine developmental trajectories and compare enrollment cohorts between 2012–2019 and 2020–2025. Additional BMI-stratified analyses were performed to explore heterogeneity in trajectory patterns.

**Results:**

Most physical fitness indicators showed continuous improvement during 2013–2019, whereas these improvements generally became attenuated after 2020. Developmental trajectories differed significantly between enrollment cohorts. Compared with the pre-2020 (2012–2019) cohorts, the post-2020 (2020–2025) cohorts generally demonstrated higher baseline levels for several indicators but weaker developmental trajectories across academic grades. BMI-stratified analyses further revealed substantial heterogeneity in developmental trajectories across BMI groups, particularly for endurance- and strength-related indicators.

**Conclusions:**

Physical fitness among university students showed substantial temporal changes between 2013 and 2025. Although the post-2020 cohorts demonstrated relatively higher baseline performance for several indicators, their subsequent developmental trajectories across university years were generally weaker than those of the pre-2020 cohorts. These findings highlight the importance of continuous physical fitness monitoring and targeted health promotion strategies tailored to different physical fitness components and BMI groups.

## 1 Introduction

University represents a critical transitional period from adolescence to adulthood. During this stage, substantial changes occur in lifestyle and health-related behaviors, which may have long-term implications for health outcomes in later life (1). However, physical inactivity is highly prevalent among university students worldwide (2). Accumulating epidemiological evidence indicates that adequate physical activity is associated with a lower risk of multiple chronic diseases (3–7). Therefore, understanding physical fitness development among university students has become an important public health concern.

Recent environmental and behavioral changes among university students may have influenced physical fitness development over time, with the COVID-19 pandemic and related containment measures further altering patterns of physical activity and daily behaviors (8). Previous studies have reported reductions in physical activity and increases in sedentary behavior during and after the pandemic period, accompanied by varying degrees of decline in physical fitness (9–14). However, most existing studies have relied on cross-sectional designs or short-term pre- and post-pandemic comparisons, which mainly reflect differences in physical fitness levels at specific time points. Consequently, long-term developmental trajectories of physical fitness among university students remain insufficiently understood.

In addition, important limitations remain in the existing literature. Most previous studies have focused on a single enrollment cohort (15–17), making it difficult to distinguish age-related developmental changes from broader temporal and environmental influences. Students from different enrollment cohorts may experience distinct educational, social, and behavioral contexts (18, 19). However, few studies have compared these cohorts within a unified longitudinal framework. As a result, the respective contributions of temporal changes and cohort differences to physical fitness development remain unclear.

Furthermore, physical fitness development is characterized by marked non-linearity and substantial inter-individual variability. Previous studies have shown that physical fitness indicators follow distinct developmental trajectories across adolescence and early adulthood (20), and that factors such as BMI may further shape these trajectories (21). Recent trajectory-based studies have also reported distinct developmental patterns in physical fitness (22). Therefore, a trajectory-based approach may help more systematically characterize the dynamic patterns of physical fitness development and enable longitudinal comparisons across multiple cohorts.

Therefore, longitudinal evidence integrating both population-level temporal trends and individual-level developmental trajectories across multiple enrollment cohorts remains limited. Using a 13-year accelerated longitudinal design, the present study aimed to examine temporal trends and developmental trajectories of physical fitness among university students between 2013 and 2025.

## 2 Materials and methods

### 2.1 Data source and participants

**Figure 1:**
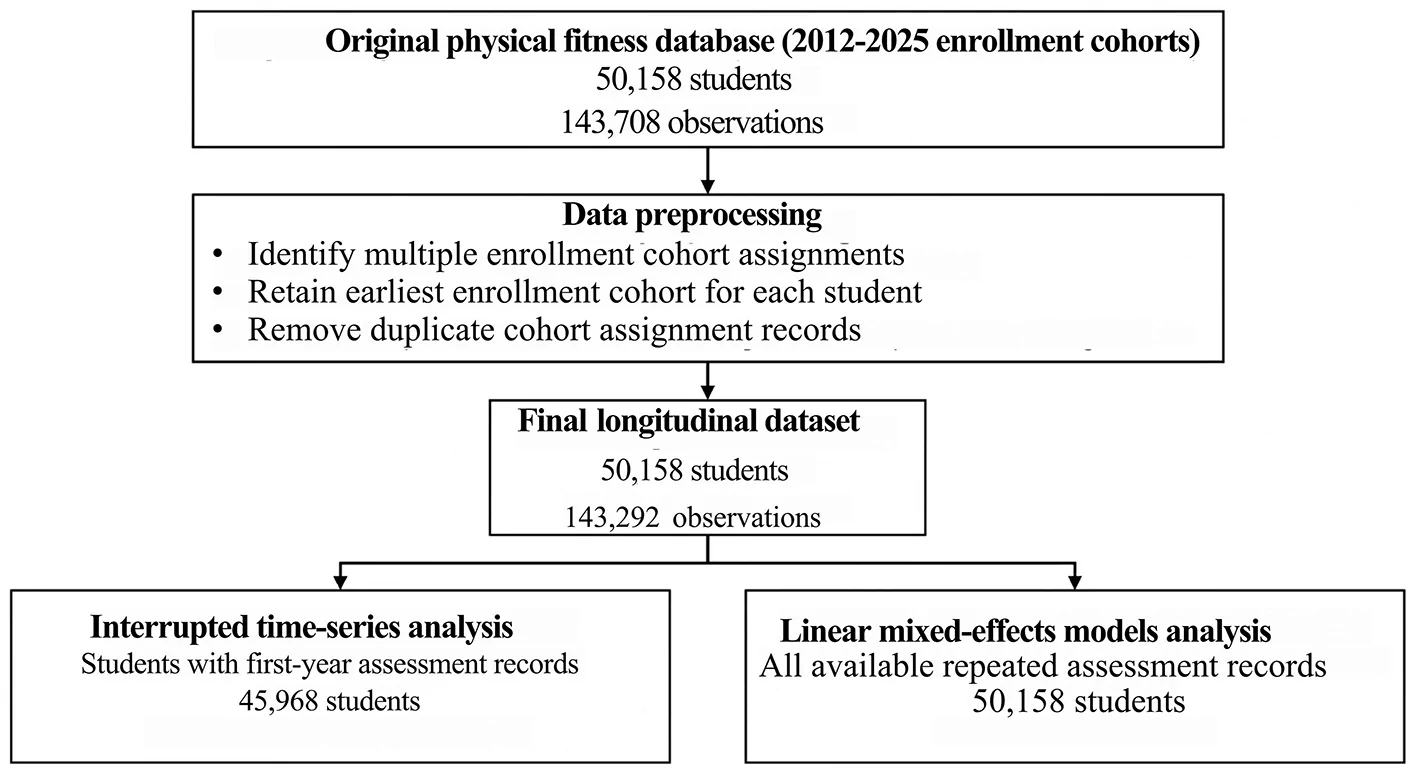
Flowchart of study population selection and longitudinal dataset construction.

This study was based on annual physical fitness monitoring data from undergraduate students at Anhui Medical University (Hefei, China) between 2013 and 2025. Annual physical fitness assessments were routinely conducted among undergraduate students during their university studies, which was typically conducted between October and November. A total of 50,158 undergraduate students were initially identified, contributing 143,708 physical fitness assessment records. During data preprocessing, 416 records were removed due to duplicate enrollment cohort assignments. After this procedure, the final longitudinal dataset included 50,158 students with 143,292 observations. The study population selection process and the construction of analytical datasets for different analyses are presented in Figure 1.

Each student contributed between one and four available repeated measurements during the study period. Although all analytical variables were complete, the number of repeated measurements varied among students because of differences in observable follow-up windows and incomplete annual participation. Among the included students, 71.4% contributed three or more repeated measurements, and 89.3% contributed at least two measurements. Only 10.7% of students contributed a single measurement, mainly from the 2023–2025 enrollment cohorts, who had not yet reached subsequent assessment opportunities. The distribution of available repeated measurements is presented in Supplementary Tables S1, S2. To reconstruct developmental trajectories across university years, partial observations from the 2012 enrollment cohort collected during 2013–2014 were also included. More recent cohorts had not yet completed the full four-year observation period at the time of data collection.

This study was approved by the Ethics Committee of Anhui Medical University (Approval No. 84240004).

### 2.2 Physical fitness measurements

The physical fitness tests were based on the National Student Physical Fitness and Health Standard of China, which has been widely used in student fitness surveillance and has demonstrated good reliability and validity (23). This study included seven physical fitness indicators: body mass index (BMI), vital capacity, 50-m dash, standing long jump, sit-and-reach, upper-body muscle strength (pull-ups for males and 1-min sit-ups for females), and endurance run (800 m for females and 1,000 m for males).

All assessments were conducted by trained physical education instructors following standardized testing procedures. Examiners received uniform training in testing methods, operational procedures, and scoring criteria. Testing equipment was routinely inspected and maintained to ensure measurement quality and consistency. All measurement data were recorded immediately after testing and underwent routine completeness and consistency checks before being incorporated into the annual physical fitness database.

#### 2.2.1 BMI

BMI was calculated as body weight (kg) divided by height squared (m^2^). According to the criteria recommended by the Working Group on Obesity in China, BMI ≥ 28.0 kg/m^2^ was defined as obesity, 24.0–27.9 kg/m^2^ as overweight, 18.5–23.9 kg/m^2^ as normal weight, and < 18.5 kg/m^2^ as underweight (24). Height and weight were measured using a standardized electronic scale.

#### 2.2.2 Vital capacity

Vital capacity was measured using a spirometer with a disposable mouthpiece. Participants were instructed to perform a maximal exhalation after full inspiration, and maximal expiratory volume was recorded in milliliters (mL).

#### 2.2.3 50-m dash

The 50-m dash test was used to assess speed performance. Participants completed one maximal sprint trial, and running time was recorded to the nearest 0.1 s.

#### 2.2.4 Sit-and-reach

Participants sat barefoot on the testing apparatus with their knees fully extended and gradually reached forward as far as possible. The maximal distance reached was recorded in centimeters.

#### 2.2.5 Standing long jump

Standing long jump performance was assessed as the maximal horizontal jump distance from a standing position. The longest distance was recorded in centimeters.

#### 2.2.6 Endurance run

Endurance run performance was assessed using the 800-m run for females and the 1,000-m run for males. Participants were instructed to complete the run as quickly as possible, and running time was recorded to the nearest 0.1 s.

#### 2.2.7 Upper-body muscle strength

Upper-body muscle strength was assessed using pull-ups for males and a 1-min sit-up test for females. Pull-ups were performed on a horizontal bar using an underhand grip, and a valid repetition required elbow flexion beyond 90 degrees. For females, the 1-min sit-up test recorded the number of correctly completed sit-ups within 60 s, with each repetition requiring the elbows to reach or pass the knees.

### 2.3 Statistical analysis

Statistical analyses were conducted at both the population and individual levels. Population-level temporal trends were examined using interrupted time-series analysis, whereas individual developmental trajectories were modeled using linear mixed-effects models. All statistical analyses were performed using R software (version 4.5.0; R Foundation for Statistical Computing, Vienna, Austria). All statistical tests were two-sided, and statistical significance was set at *p* < 0.05. To ensure consistent directional interpretation across physical fitness indicators, performance in the 50-m dash and 800/1,000-m endurance runs was converted to running speed (m/s), with higher values indicating better physical fitness.

#### 2.3.1 Baseline comparisons

Baseline characteristics of first-year students were compared between the pre-2020 and post-2020 cohorts, restricted to students with available first-year assessments. Continuous variables were compared using independent-samples *t*-tests, and categorical variables were compared using χ^2^ tests. Standardized mean differences (SMDs) were calculated to assess between-group differences independent of sample size. Given the large sample size, conventional hypothesis tests may identify trivial differences as statistically significant. SMD values of 0.2, 0.5, and 0.8 were interpreted as representing small, moderate, and large differences, respectively (25, 26).

#### 2.3.2 Interrupted time-series analysis

Interrupted time-series analyses were conducted using annual cohort means of first-year students to examine population-level temporal trends in physical fitness across enrollment years. Analyses were based on annual physical fitness monitoring data from first-year students between 2013 and 2025, with yearly mean values of each physical fitness indicator treated as the unit of observation. Based on the timing of major changes in campus management and educational activities at Anhui Medical University, 2020 was specified a priori as the interruption point, as it represented a transition period characterized by substantial changes in campus learning environments, physical education delivery, and opportunities for physical activity.

Beginning in late January 2020, the university implemented campus COVID-19 prevention measures. During the spring semester of 2020, teaching activities were delivered online and physical education courses were conducted remotely. Students gradually returned to campus from May 2020; however, restrictions affecting campus activities remained. Additional temporary control measures were implemented in spring 2022 during the Omicron outbreak, but these were considered part of the continuing post-pandemic adjustment period rather than a new interruption point. Following the optimization of national COVID-19 policies in December 2022, campus activities gradually returned to normal.

A segmented regression model was applied to evaluate changes in the level and trend of physical fitness indicators before and after the predefined interruption point (2020) (27, 28). The following segmented regression model was constructed:


$$\begin{array}{l} Y_{t}=\beta_{0}+\beta_{1}T_{t}+\beta_{2}X_{t}+\beta_{3}P_{t}+\in_{t} \end{array}$$

where *Y*_*t*_ represents the annual mean value of the physical fitness indicator among first-year students in year *t*; *T*_*t*_ denotes the number of years since 2013 (2013 = 0, 2014 = 1, and so forth); *X*_*t*_ represents the time indicator for the 2020 interruption point (0 for years before 2020 and 1 for 2020 and thereafter); *P*_*t*_ denotes the time elapsed since 2020 (0 for years before 2020, 1 for 2021, 2 for 2022, and so forth); β_1_ estimated the underlying temporal trend in physical fitness during the pre-pandemic period (2013–2019); β_2_ estimated the immediate level change at the predefined interruption point (2020) and was used to assess whether an abrupt deviation from the pre-2020 trend occurred; β_3_ estimated the change in the post-2020 trend relative to the pre-pandemic trend and was used to assess whether the post-2020 trend differed from the pre-2020 trend; ϵ_*t*_ denotes the random error term.

To account for potential autocorrelation and heteroscedasticity, Newey–West heteroscedasticity and autocorrelation consistent (HAC) standard errors were applied (29). Given the annual time-series data and the limited number of observations (13 time points), a lag length of 1 was specified with reference to the recommendations of Newey and West (1994) (30). The Durbin–Watson statistic was additionally calculated to assess first-order serial correlation, as recommended in interrupted time-series analyses (28).

#### 2.3.3 Longitudinal trajectory modeling

Individual-level analyses were conducted using an accelerated longitudinal design (31). Repeated observations from different enrollment cohorts were aligned along a unified academic-grade time scale to reconstruct physical fitness developmental trajectories. Because the database covered the period from 2013 to 2025, different enrollment cohorts had different observable follow-up windows, resulting in varying numbers of repeated measurements across participants. To improve reconstruction of developmental trajectories, partial observations from the 2012 enrollment cohort were included. Because the database did not contain fourth-year physical fitness assessment records for the 2012 enrollment cohort, only second- and third-year observations from this cohort were available for trajectory reconstruction.

Linear mixed-effects models were used to model physical fitness developmental trajectories (32, 33). Models were adjusted for age and assessment year to account for age-related differences and secular temporal trends. Participants were classified according to enrollment period as pre-2020 and post-2020 cohorts. Cohort effects and cohort-by-grade interactions were included in the models to assess differences in developmental trajectories. All physical fitness indicators were standardized into sex-specific Z-scores to ensure comparability across indicators with different measurement scales. Because baseline BMI served as the stratification variable in subsequent analyses, BMI itself was not modeled as a longitudinal outcome. Models included a random intercept for each participant to account for within-subject correlation and random slopes for academic grade to capture individual differences in longitudinal change. Random-effects structures were compared using Akaike information criterion (AIC) and likelihood ratio tests (LRT), and optimal models were selected based on model fit indices (Supplementary Tables S3, S4). Parameters were estimated using restricted maximum likelihood (REML).

Given the unbalanced longitudinal structure arising from cohort-specific follow-up windows and occasional missing annual assessments, linear mixed-effects models were particularly appropriate for this study. This approach incorporated all available repeated measurements for parameter estimation without requiring listwise deletion of participants with incomplete follow-up data. To assess the potential for selection bias related to incomplete follow-up, baseline characteristics were compared between students who contributed four physical fitness assessments and those who contributed fewer than four assessments among enrollment cohorts with available four-year observation windows (2015–2022 cohorts). Standardized mean differences (SMDs) for all key baseline indicators were below 0.2, indicating good comparability between groups and suggesting that substantial selection bias due to incomplete follow-up was unlikely, as shown in Supplementary Table S5.

Exploratory change-point analyses using segmented regression were additionally conducted to identify potential data-driven temporal changes in physical fitness trends across the study period. These analyses used the same annual cohort-level mean values as the interrupted time-series analyses and were performed separately by sex for each fitness indicator. The segmented regression models were fitted following the approach for estimating regression models with unknown break-points (34). The estimated change points were compared with the predefined 2020 interruption point to assess whether data-driven temporal transitions were consistent with the primary ITS specification.

#### 2.3.4 BMI-stratified analyses

To examine trajectory differences across BMI categories on trajectory differences, participants were classified into BMI groups based on baseline BMI at university entry. Interaction terms between BMI group, academic grade, and cohort were included in the linear mixed-effects models to examine heterogeneity in physical fitness developmental trajectories across BMI categories.

## 3 Results

### 3.1 Baseline characteristics of incoming university students

Table 1 presents the baseline characteristics of 45,982 first-year students included in the baseline comparison analyses, including 22,712 students from the pre-2020 enrollment cohorts (2013–2019) and 23,270 students from the post-2020 cohorts (2020–2025).

**Table 1:** Baseline characteristics of first-year university students in the pre-2020 and post-2020 cohorts.

Variables	Pre-2020 cohort (*n* = 22,712)	Post-2020 cohort (*n* = 23,270)	*p* value	SMD
Age (years)	18.71 (1.11)	18.40 (0.66)	< 0.001	0.337
Gender, *n* (%)			< 0.001	0.037
Male	10,759 (47.4)	10,594 (45.5)		
Female	11,953 (52.6)	12,676 (54.5)		
BMI (kg/m^2^)	21.22 (3.17)	22.21 (3.53)	< 0.001	0.295
Weight category, *n* (%)			< 0.001	0.281
Underweight	4,046 (17.8)	2,261 (9.7)		
Normal	14,981 (66.0)	15,497 (66.6)		
Overweight	2,799 (12.3)	3,937 (16.9)		
Obesity	886 (3.9)	1,575 (6.8)		
Physical fitness indicators in males				
1,000-m run (m/s)	3.99 (0.44)	4.04 (0.52)	< 0.001	0.105
50-m dash (m/s)	6.59 (0.48)	6.64 (0.48)	< 0.001	0.092
Standing long jump (cm)	224.51 (21.11)	223.57 (22.90)	0.002	0.043
Vital capacity (mL)	4,043.25 (716.77)	4,390.80 (676.00)	< 0.001	0.499
Sit-and-reach (cm)	14.86 (7.11)	17.43 (6.54)	< 0.001	0.377
Pull-ups (repetitions)	4.37 (4.10)	4.66 (5.29)	< 0.001	0.06
Physical fitness indicators in females				
800-m run (m/s)	3.35 (0.35)	3.34 (0.39)	0.002	0.04
50-m dash (m/s)	5.37 (0.40)	5.42 (0.38)	< 0.001	0.122
Standing long jump (cm)	169.76 (16.82)	169.73 (17.64)	0.907	0.001
Vital capacity (mL)	2,701.38 (540.88)	3,081.92 (469.94)	< 0.001	0.751
Sit-and-reach (cm)	18.18 (6.17)	21.71 (5.53)	< 0.001	0.603
Sit-ups (repetitions)	28.74 (8.95)	35.63 (9.51)	< 0.001	0.746

Data are presented as mean (SD) or *n* (%). SMD = standardized mean difference. Baseline differences between cohorts were assessed using independent-samples *t*-tests for continuous variables and χ^2^ tests for categorical variables. Standardized mean differences (SMDs) were additionally calculated to evaluate the magnitude of between-group differences. SMD values of 0.2, 0.5, and 0.8 indicate small, moderate, and large differences, respectively. The pre-2020 cohort included students enrolled between 2012 and 2019, whereas the post-2020 cohort included students enrolled between 2020 and 2025. All between-group comparisons were statistically significant (*p* < 0.05), except for female standing long jump performance.

Compared with the pre-2020 cohorts, students in the post-2020 cohorts were younger and had higher BMI levels at university entry. The post-2020 cohorts also showed shifts in BMI category distribution, characterized by higher proportions of overweight and obesity and a lower proportion of underweight students.

Differences in baseline physical fitness indicators were observed between cohorts, with several indicators showing higher levels in the post-2020 cohorts. These differences were particularly evident for vital capacity and sit-and-reach performance.

Sex-stratified analyses showed more pronounced cohort differences among female students, especially for upper-body muscle strength, vital capacity, and sit-and-reach performance. Similar but smaller differences were also observed among male students for vital capacity and flexibility indicators.

By contrast, cohort differences in 50-m dash and endurance run performance were generally small. Standing long jump performance showed minimal differences between cohorts, with no significant cohort difference observed among female students (*p* = 0.907).

### 3.2 Temporal trends in physical fitness among incoming cohorts

**Figure 2:**
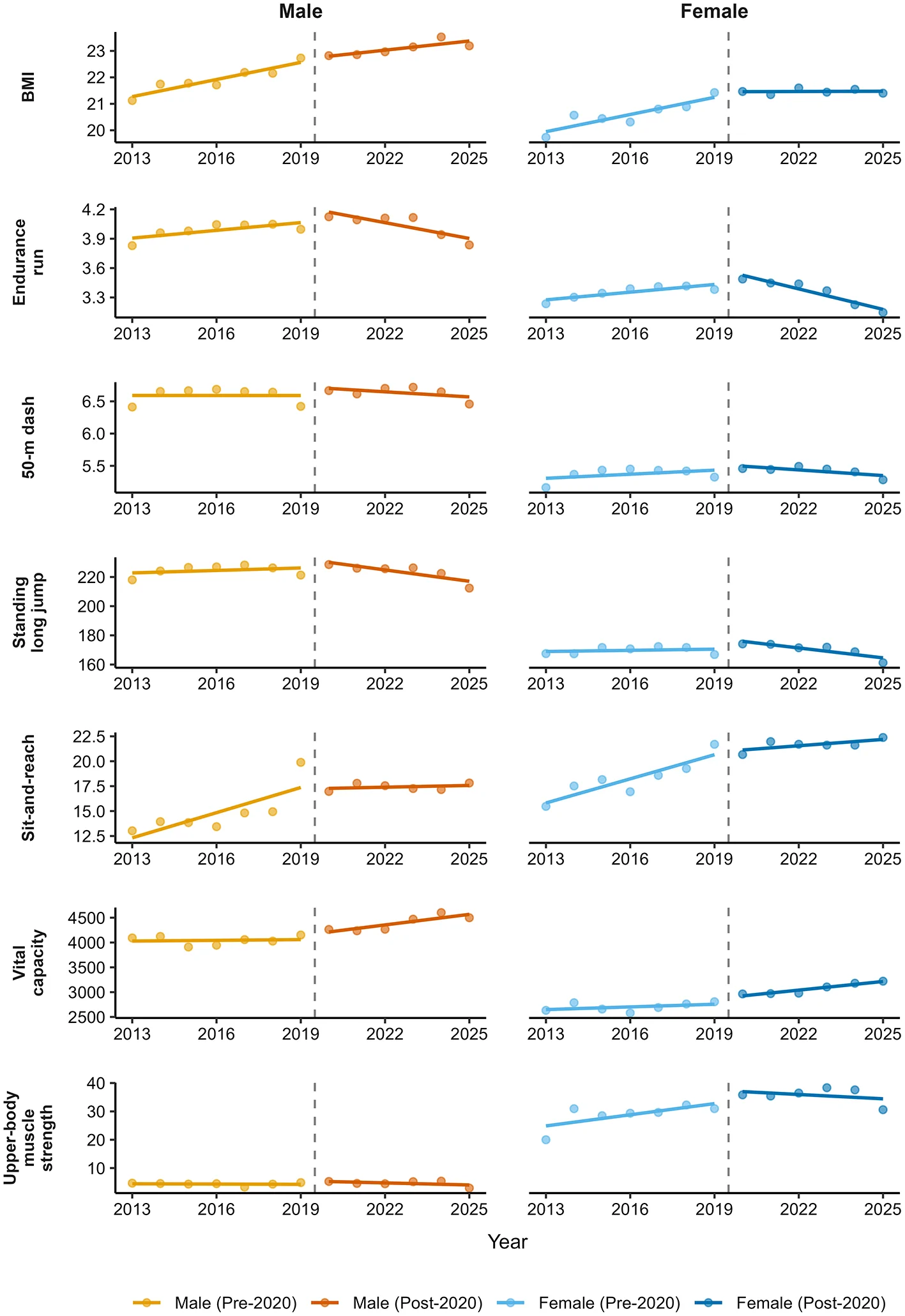
Interrupted time-series plots of physical fitness indicators among first-year university students, 2013–2025. The figure is arranged as a matrix with sex displayed in columns and fitness indicators in rows. Points represent observed cohort means, and solid lines represent segmented regression fits for the pre-2020 (2013–2019) and post-2020 (2020–2025) periods. The vertical dashed line indicates the 2020 interruption. Units: BMI (kg/m^2^), 50-m dash and endurance run (m/s), vital capacity (mL), standing long jump (cm), sit-and-reach (cm), and upper-body muscle strength (repetitions).

After accounting for long-term temporal trends, interrupted time-series analyses identified significant changes in level and slope around the 2020 time point for several physical fitness indicators, as illustrated in Figure 2 and summarized in Table 2.

**Table 2:** Segmented regression estimates from interrupted time series analyses of physical fitness indicators among university students, 2013–2025.

Fitness indicator	Gender	β_1_ (95% CI)	β_2_ (95% CI)	β_3_ (95% CI)	Durbin-Watson	*p* value
BMI	Male	**0.216** (0.161, 0.271)	0.015 (−0.206, 0.235)	**−0.101** (−0.182, −0.020)	2.64	0.604
	Female	**0.217** (0.143, 0.290)	−0.004 (−0.277, 0.269)	**−0.214** (−0.293, −0.134)	2.51	0.500
Endurance run	Male	**0.026** (0.003, 0.050)	0.081 (−0.030, 0.192)	**−0.080** (−0.121, −0.039)	1.26	0.002
	Female	**0.026** (0.011, 0.042)	0.068 (−0.010, 0.146)	**−0.096** (−0.123, −0.069)	1.11	< 0.001
50-m dash	Male	0.000 (−0.056, 0.055)	0.111 (−0.125, 0.347)	−0.026 (−0.097, 0.044)	1.45	0.009
	Female	0.021 (−0.021, 0.063)	0.041 (−0.122, 0.204)	**−0.050** (−0.102, 0.002)	1.19	0.001
Standing long jump	Male	0.563 (−1.064, 2.189)	3.298 (−3.926, 10.521)	**−3.15**7 (−5.525, −0.788)	1.28	0.002
	Female	0.266 (−0.744, 1.276)	**5.102** (0.546, 9.658)	**−2.535** (−4.213, −0.856)	1.54	0.017
Sit-and-reach	Male	**0.841** (0.232, 1.451)	−0.928 (−4.301, 2.444)	**−0.783** (−1.355, −0.210)	2.20	0.258
	Female	**0.807** (0.521, 1.093)	−0.332 (−2.087, 1.422)	**−0.595** (−0.859, −0.331)	2.35	0.364
Vital capacity	Male	4.9 (−27.4, 37.2)	**149.1** (21.7, 276.6)	**66.1** (18.7, 113.4)	1.81	0.066
	Female	17.9 (−4.2, 40.1)	**150.3** (50.6, 250.0)	**40.2** (11.1, 69.3)	1.77	0.056
Upper-body muscle strength	Male	−0.031 (−0.213, 0.151)	1.017 (−0.174, 2.208)	−0.214 (−0.600, 0.172)	2.02	0.151
	Female	**1.311** (0.176, 2.446)	2.925 (−0.901, 6.752)	**−1.818** (−3.546, −0.089)	1.98	0.131

Values are unstandardized regression coefficients with 95% confidence intervals (CIs) in parentheses. Statistically significant estimates (*p* < 0.05) are shown in bold. β_1_ represents the pre-2020 annual trend (2013–2019); β_2_ represents the immediate level change in 2020; β_3_ represents the change in annual trend after 2020 relative to the pre-2020 trend. Newey–West HAC standard errors (lag = 1) were used for all interrupted time series models. Residual autocorrelation was assessed using the Durbin–Watson statistic and its corresponding *p* value.

During 2013–2019, most physical fitness indicators showed gradual improvements across incoming cohorts. Endurance-related indicators increased steadily in both sexes, with annual increases of 0.026 m/s in both males and females. Flexibility showed significant positive trends, whereas power-related indicators exhibited upward but non-significant trends during this period. BMI increased continuously in both sexes, whereas no significant pre-2020 trends were observed for male 50-m dash performance, vital capacity, or upper-body muscle strength.

Around 2020, significant immediate level changes were observed for female standing long jump performance and vital capacity in both sexes. Standing long jump performance increased in both sexes, by 3.298 cm in males and 5.102 cm in females, although statistical significance was observed only in females. Vital capacity increased by 149.1 mL in males and 150.3 mL in females.

During 2020–2025, improvements in most physical fitness indicators became attenuated compared with the pre-2020 period. Endurance-related indicators showed the most pronounced reductions in slope, with annual changes decreasing by 0.080 m/s in males and 0.096 m/s in females relative to the pre-2020 trends. Similar attenuation patterns were also observed for standing long jump performance (−3.157 cm/year in males and −2.535 cm/year in females) and sit-and-reach performance (−0.783 cm/year in males and −0.595 cm/year in females), whereas upper-body muscle strength showed a significant attenuation only among females (−1.818 repetitions/year). In contrast, vital capacity continued to increase during the post-2020 period, with annual increases of 66.1 mL/year in males and 40.2 mL/year in females.

Overall, these findings suggest that temporal changes in physical fitness among incoming university cohorts were characterized more by slowing or attenuation of previously improving trends than by uniform declines across all indicators.

Exploratory change-point analyses identified potential temporal change points across fitness indicators (Supplementary Table S6). Change points for BMI were identified in 2020 for both males and females, consistent with the predefined interruption point used in the interrupted time-series analysis. Change points for sit-and-reach performance were identified in 2019 among males and 2021 among females, whereas endurance performance showed later change points in males (2023) and females (2022). No potential change points were identified for standing long jump, vital capacity, 50-m dash, or upper-body muscle strength.

### 3.3 Developmental trajectories of physical fitness across university years

**Figure 3:**
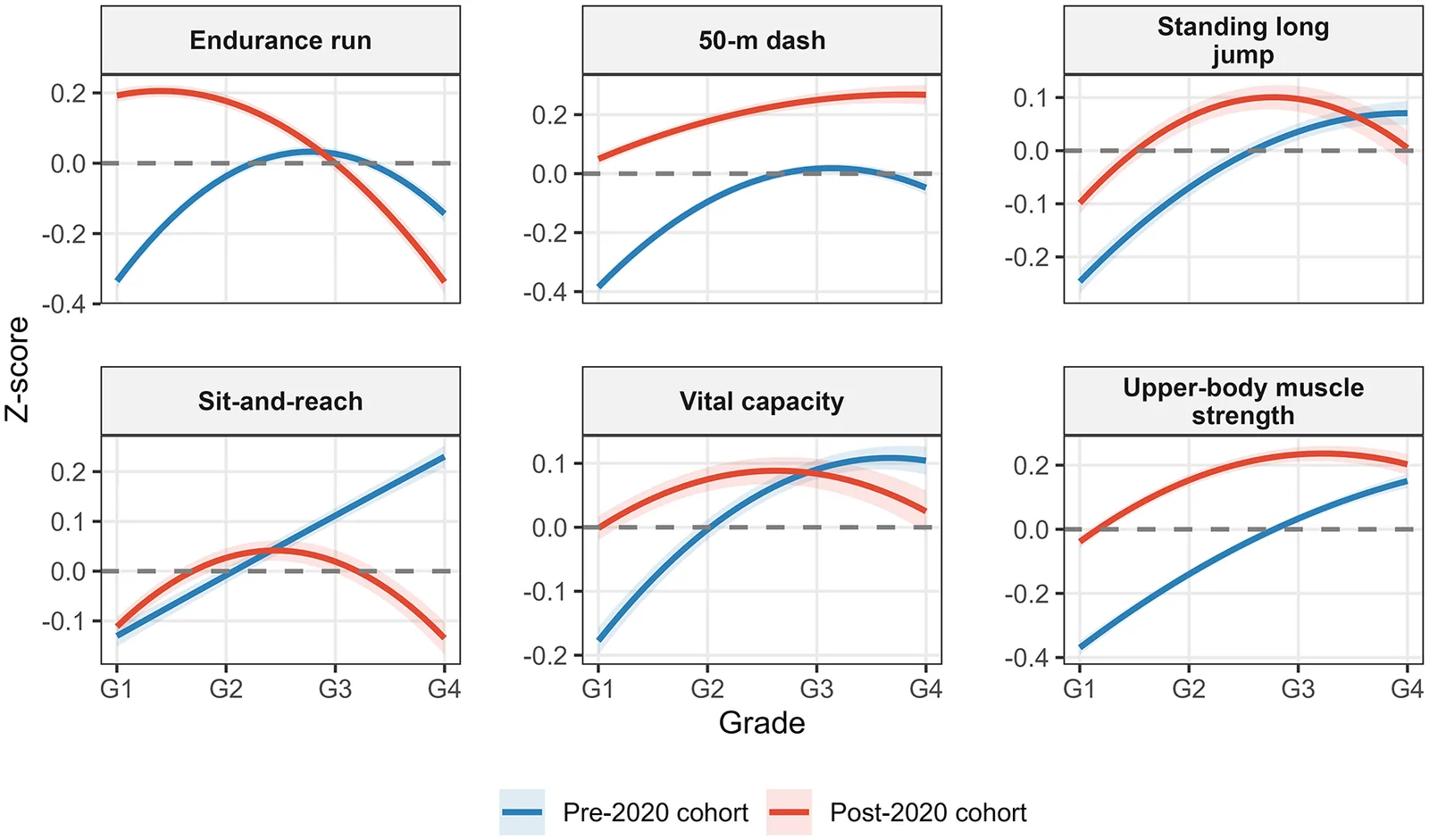
Estimated longitudinal trajectories of physical fitness indicators across university grades. Solid lines represent estimated marginal means, and shaded areas indicate 95% confidence intervals. Blue and red lines represent pre-2020 and post-2020 cohorts, respectively. G1–G4 denote academic grades 1–4. All outcomes were standardized as sex-specific *Z*-scores (higher values indicate better performance).

Linear mixed-effects models identified significant cohort differences in developmental trajectories, which were illustrated by the estimated marginal means shown in Figure 3 and summarized in Table 3.

**Table 3:** Fixed-effect estimates from linear mixed-effects models of physical fitness trajectories.

Source of variance	Fixed-effect estimate	Standard error	*Z*-value
Endurance run			
Intercept	−0.327	0.014	**−23.08**
Age	−0.025	0.004	**−6.43**
Assessment year	−0.070	0.002	**−37.71**
Grade	0.621	0.013	**48.03**
Grade^2^	−0.109	0.003	**−41.02**
Post-2020 cohort	0.812	0.024	**34.33**
Grade × Cohort	−0.346	0.019	**−18.27**
Grade^2^ × Cohort	0.015	0.004	**3.62**
50-m dash			
Intercept	−0.435	0.014	**−31.24**
Age	−0.033	0.004	**−7.85**
Assessment year	−0.054	0.002	**−27.71**
Grade	0.536	0.012	**43.20**
Grade^2^	−0.082	0.003	**−32.82**
Post-2020 cohort	0.702	0.024	**29.68**
Grade × Cohort	−0.310	0.018	**−17.29**
Grade^2^ × Cohort	0.051	0.004	**13.12**
Standing long jump			
Intercept	−0.312	0.013	**−24.30**
Age	−0.025	0.004	**−5.77**
Assessment year	−0.022	0.002	**−10.54**
Grade	0.283	0.011	**25.12**
Grade^2^	−0.034	0.002	**−15.46**
Post-2020 cohort	0.092	0.023	**4.03**
Grade × Cohort	0.079	0.016	**4.96**
Grade^2^ × Cohort	−0.031	0.004	**−8.93**
Sit-and-reach			
Intercept	−0.681	0.013	–**54.67**
Age	−0.007	0.004	−1.77
Assessment year	0.069	0.002	**35.32**
Grade	0.109	0.011	**10.09**
Grade^2^	0.003	0.002	1.39
Post-2020 cohort	−0.151	0.022	**−6.87**
Grade × Cohort	0.239	0.015	**15.59**
Grade^2^ × Cohort	−0.073	0.003	**−21.98**
Upper-body muscle strength			
Intercept	−0.724	0.013	**−54.44**
Age	−0.013	0.004	**−3.17**
Assessment year	0.013	0.002	**6.93**
Grade	0.306	0.012	**25.58**
Grade^2^	−0.024	0.002	**−9.77**
Post-2020 cohort	0.275	0.023	**12.12**
Grade × Cohort	0.089	0.017	**5.15**
Grade^2^ × Cohort	−0.041	0.004	**−10.75**
Vital capacity			
Intercept	−0.886	0.013	**−69.69**
Age	0.003	0.004	0.76
Assessment year	0.068	0.002	**34.99**
Grade	0.294	0.011	**26.43**
Grade^2^	−0.039	0.002	**−17.82**
Post-2020 cohort	0.271	0.022	**12.18**
Grade × Cohort	−0.106	0.016	**−6.68**
Grade^2^ × Cohort	0.004	0.003	1.03

Intercept estimates represent the estimated physical fitness level at the first academic year. All fitness outcomes were standardized as sex-specific *Z*-scores, with higher values indicating better physical fitness performance. Running performance was converted from completion time to speed (m/s) before standardization so that higher values consistently indicate better performance. Age and assessment year were included as covariates in all models. Bold values indicate statistically significant estimates ( *p* < 0.05).

For endurance-related indicators, the 2020–2025 cohort had higher estimated baseline physical fitness levels at university entry (β = 0.812, SE = 0.024), but a steeper decline across academic grades compared with the 2012–2019 cohort (β = −0.346, SE = 0.019). A small positive quadratic interaction was also observed (β = 0.015, SE = 0.004), suggesting a slight curvature in the trajectory over time.

Speed-related indicators showed a similar pattern. The post-2020 cohort had higher estimated baseline physical fitness levels (β = 0.702, SE = 0.024), but a weaker linear change across academic grades (β = −0.310, SE = 0.018), together with a positive quadratic interaction (β = 0.051, SE = 0.004), indicating a more attenuated developmental trajectory.

For power-related indicators, the 2020–2025 cohort exhibited slightly higher estimated baseline physical fitness levels (β = 0.092, SE = 0.023) and a greater linear increase across academic grades (β = 0.079, SE = 0.016), but this improvement slowed over time, as indicated by a negative quadratic interaction (β = −0.031, SE = 0.003).

Upper-body muscle strength demonstrated higher estimated baseline physical fitness levels in the post-2020 cohort (β = 0.275, SE = 0.023) and a greater linear increase across academic grades (β = 0.089, SE = 0.017), but with a decelerating pattern in later years (β = −0.041, SE = 0.004).

Flexibility showed lower baseline levels in the 2020–2025 cohort (β = −0.151, SE = 0.022), with a greater linear increase across academic grades (β = 0.239, SE = 0.015) and a negative quadratic term (β = −0.073, SE = 0.003), initial increase followed by deceleration.

Vital capacity presented higher estimated baseline physical fitness levels in the post-2020 cohort (β = 0.271, SE = 0.022), but a weaker linear increase across grades (β = −0.106, SE = 0.016).

Age effects were observed across several physical fitness indicators. After adjusting for cohort and temporal effects, age was negatively associated with endurance run performance (β = −0.025, SE = 0.004), 50-m dash performance (β = −0.033, SE = 0.004), and standing long jump performance (β = −0.025, SE = 0.004). No significant age effects were observed for upper-body muscle strength, vital capacity, or flexibility.

In contrast, assessment year showed stronger and more consistent associations across physical fitness indicators. Significant negative associations were observed for endurance run (β = −0.070, SE = 0.002), 50-m dash (β = −0.054, SE = 0.002), and standing long jump (β = −0.022, SE = 0.002). Positive associations were observed for flexibility (β = 0.069, SE = 0.002), vital capacity (β = 0.068, SE = 0.002), and upper-body muscle strength (β = 0.013, SE = 0.002).

Overall, students entering university after 2020 generally showed higher baseline performance but weaker developmental trajectories across several physical fitness indicators than those entering before 2020.

### 3.4 BMI-related differences in developmental trajectories

BMI-stratified analyses revealed heterogeneous physical fitness trajectories across enrollment cohorts and academic grades, with main effects and interaction effects summarized in Supplementary Table S7.

For endurance-related indicators, trajectory differences across BMI categories were evident. The underweight group showed a weaker developmental trajectory within the 2020–2025 cohort (β = −0.076, SE = 0.014), whereas positive interaction effects were observed in the overweight (β = 0.036, SE = 0.013) and obese groups (β = 0.111, SE = 0.021), indicating divergent developmental patterns compared with the normal-weight group.

For speed-related indicators, BMI-related interaction effects were relatively small. The underweight group showed a negative interaction effect (β = −0.045, SE = 0.014), while weak positive effects were observed in the overweight (β = 0.022, SE = 0.012) and obese groups (β = 0.031, SE = 0.020).

For upper-body muscle strength, Significant three-way interactions were observed for upper-body muscle strength across all BMI groups: underweight (β = −0.035, SE = 0.013), overweight (β = 0.050, SE = 0.012), and obese (β = 0.089, SE = 0.019).

For vital capacity, significant three-way interactions were found in the overweight (β = 0.032, SE = 0.011) and obese groups (β = 0.037, SE = 0.017), but not in the underweight group. For flexibility, only the underweight group showed a significant negative interaction (β = −0.033, SE = 0.012), whereas the overweight and obese groups were not significant.

The model-estimated longitudinal trajectories of endurance run and upper-body muscle strength stratified by BMI categories are presented in Supplementary Figures S1, S2, respectively.

## 4 Discussion

### 4.1 Temporal changes in physical fitness development

This study used longitudinal physical fitness data collected from 2013 to 2025 to examine temporal changes in university students' physical fitness from both population and developmental perspectives. At the population level, most physical fitness indicators showed gradual improvement across incoming cohorts during 2012–2019. Around 2020, several indicators exhibited significant level changes, and during 2020–2025, changes in most indicators showed attenuation relative to the pre-2020 trends, although the direction and magnitude of these changes varied across indicators. For example, vital capacity continued to show a more favorable annual increase during the post-2020 period, whereas BMI continued to increase but at a slower rate. Consistent patterns were also observed in the developmental trajectory analysis: students enrolled between 2020 and 2025 generally exhibited higher estimated baseline physical fitness levels across several indicators than those enrolled between 2012 and 2019, but showed smaller or attenuated improvements throughout the university years, with some indicators showing a decelerating or downward trajectory in later academic grades.

These findings suggest that temporal differences between enrollment cohorts were reflected not only in physical fitness at university entry but also in developmental trajectories throughout the university years. Exploratory change-point analyses further indicated that temporal changes were not uniform across fitness indicators. Although BMI showed change points in 2020 for both sexes, consistent with the predefined interruption point specified in the interrupted time-series analysis, other indicators showed different potential change points or no identifiable change points. These findings suggest that temporal changes may have occurred at different stages across fitness indicators. The identified change points did not necessarily correspond to immediate changes at the predefined 2020 interruption point, because changes in the underlying temporal trajectory may occur without an abrupt level shift. For example, BMI showed a potential change point in 2020, whereas the interrupted time-series analysis suggested that this change was mainly reflected in a slower increase after 2020 rather than an immediate level change at that time point. This pre-2020 improving trend contrasts with an earlier study of Chinese male first-year college students, which reported a general decline in health-related physical fitness from 2013 to 2019 (35); this discrepancy may reflect differences in sample characteristics, regional context, or the specific fitness indicators examined, highlighting the heterogeneity of secular trends across Chinese university populations. Therefore, when evaluating long-term changes observed after 2020, it is important to distinguish baseline cohort differences from longitudinal developmental changes. A longitudinal study of German primary school children similarly reported that pre-existing secular trends may account for part of the observed differences between pandemic and pre-pandemic cohorts, particularly for lower-limb power and balance-related indicators, which were paradoxically higher in the pandemic cohorts (36), underscoring the importance of accounting for long-term background trends when interpreting period-specific changes. Consistent with this, a decade-long study from Anhui Province reported that overall physical fitness scores among college students reached a peak around 2019 before declining thereafter, alongside a long-term upward trend in overweight and obesity prevalence that predated the pandemic (37), suggesting that part of the attenuation observed after 2020 in the present study may reflect the continuation of pre-existing secular trends rather than pandemic effects alone. A previous longitudinal study also reported heterogeneous changes across different physical fitness components (38). In addition, longitudinal and historical comparison studies involving adolescents and young adults have reported declines in multiple physical fitness indicators and reduced achievement of fitness standards in recent years (39, 40)

Lifestyle changes reported during the post-2020 period may partly explain the attenuated developmental trajectories observed in the present study. A growing body of evidence has documented substantial lifestyle changes during the post-2020 period. Among university students, a systematic review reported reductions in walking, moderate-to-vigorous physical activity, and total physical activity across different countries (41), while a study of Italian medical students additionally observed increased sedentary time and altered sleep duration (42). Similar patterns have also been reported in broader populations. A scoping review found reduced physical activity together with increased sedentary and screen time among children, adolescents, and adults (43), whereas a systematic review of children and adolescents consistently reported reduced physical activity but found less consistent evidence regarding changes in sleep and dietary behaviors (44). Taken together, these behavioral changes reported during the post-2020 period may have reduced opportunities for regular habitual physical activity, thereby contributing to slower improvements in physical fitness throughout the university years. Evidence on the relationship between sedentary behavior and cardiorespiratory fitness provides additional mechanistic support for this interpretation (45).

Collectively, these findings suggest that the changes observed after 2020 may reflect a combination of longer-term lifestyle changes, broader environmental influences, and underlying secular trends, rather than temporary disruptions alone.

### 4.2 Differential developmental patterns across physical fitness components

Different physical fitness components exhibited distinct developmental patterns during university years, indicating that physical fitness development is multidimensional rather than uniform across components. These findings are consistent with previous studies reporting that recent changes in physical fitness among university students are not uniform across different components. A five-year longitudinal study spanning two consecutive COVID-19 outbreaks similarly found that fitness components were affected differently, with endurance and sprint performance declining while vital capacity, flexibility, and muscle strength improved by 2023 (38). For example, declines in cardiorespiratory endurance have also been documented in Chinese university populations (17), and repeated-measures studies have also reported deteriorations in endurance running performance across academic years (46), and a pattern similarly observed among college women, with declines in 800-m running and sit-up performance following pandemic-related lockdown; notably, women in the highest baseline fitness quartile showed larger declines in both indicators, whereas those in the lowest baseline quartile showed a smaller decline in running performance and even an improvement in sit-up performance (47). In parallel, reductions in moderate-to-vigorous physical activity and increases in sedentary behavior among adolescents and young adults have been widely documented, which is consistent with the observed trends in endurance-related indicators in the present study (48, 49).

From a mechanistic perspective, these heterogeneous patterns may reflect differential physiological sensitivity of fitness components to sustained changes in physical activity and lifestyle behaviors. Endurance-related performance appears to be particularly sensitive to habitual physical activity, and reduced cardiorespiratory fitness has similarly been observed following sustained reductions in physical activity during the pandemic period (50, 51). Previous studies have indicated that reduced physical activity and increased sedentary behavior directly affect cardiorespiratory fitness (52), and behavioral changes during the pandemic period have also been associated with alterations in sleep patterns among Chinese college students (53). By contrast, speed and upper-body muscle strength are more closely associated with neuromuscular function and may therefore exhibit greater stability, with male students showing no significant change in sprint and pull-up performance over the study period, in contrast to more pronounced changes observed in other components and among female students (54).

Taken together, the present results suggest that different physical fitness components respond differently to long-term environmental and behavioral changes during university years. These findings further indicate that physical fitness development among university students should be considered a multidimensional process rather than a single, uniform outcome.

### 4.3 BMI-related differences in physical fitness trajectories

BMI-stratified analyses revealed developmental heterogeneity in physical fitness trajectories among the 2020–2025 cohort. Compared with normal-weight students, underweight students exhibited attenuated or negative developmental trajectories across endurance-, speed-, and strength-related indicators, whereas overweight and obese students showed comparatively more favorable trajectories for endurance and muscle strength, although the corresponding effects for speed-related indicators were small and inconsistent. By contrast, no consistent BMI-related divergence was observed for vital capacity or flexibility, suggesting that this heterogeneity was not uniform across all fitness components.

These findings suggest that underweight students may be particularly vulnerable to attenuated physical fitness development during the post-2020 period, which may be related to lower lean body mass and reduced muscular fitness reserves associated with underweight status. Conversely, the relatively more favorable trajectories observed among overweight and obese students for endurance and strength-related indicators may reflect a “catch-up” pattern, whereby students with lower baseline fitness may have had greater room for improvement as campus activities gradually normalized after 2020. This interpretation is consistent with cross-sectional evidence indicating that, although overweight and obese students consistently show lower absolute fitness levels—particularly for strength- and endurance-related indicators—than their normal-weight peers, the direction of change over time may differ from the direction of baseline differences (55).

It should also be noted that BMI-based classification may not fully capture underlying differences in body composition. Previous research on Chinese university students has shown that even individuals within the normal BMI range can exhibit poorer physical fitness if they have elevated body fat and reduced skeletal muscle mass, a phenotype known as normal-weight obesity (56), with the associations partially mediated by lower skeletal muscle mass. This suggests that muscular fitness and body composition, rather than BMI category alone, may be an important determinant of physical fitness trajectories, and that some of the heterogeneity observed across BMI groups in the present study could partly reflect underlying differences in body composition that were not captured by BMI classification. Previous longitudinal studies have similarly identified baseline physical fitness as an important determinant of subsequent physical fitness development across BMI categories (57).

The divergent trajectories observed across BMI groups after 2020 may also reflect differential recovery patterns following pandemic-related disruptions. A five-year longitudinal study spanning two consecutive COVID-19 outbreaks among Chinese college students reported that underweight and obese students showed gradual improvement in total fitness scores over time, whereas normal-weight and overweight students experienced initial declines followed by later recovery (38), a pattern broadly consistent with the more favorable post-2020 trajectories observed among overweight and obese students in the present study. A population-wide cohort study of Slovenian children similarly found that, although overall fitness remained below pre-pandemic levels through 2022 across most weight groups, boys with obesity showed comparatively greater recovery than their normal-weight and overweight peers (58), further suggesting that post-pandemic developmental trajectories may not follow a uniform pattern across weight categories.

Overall, the present findings suggest that BMI-related heterogeneity was reflected not only in baseline physical fitness levels but also in subsequent developmental trajectories throughout the university years, with underweight students showing consistently less favorable patterns and overweight or obese students showing a relative catch-up in endurance and strength. These differences may arise from a combination of baseline disparities, underlying body composition differences not captured by BMI classification, and differential responses to environmental and behavioral changes observed after 2020, although the specific behavioral and physiological mechanisms remain to be elucidated in future research.

### 4.4 Implications and limitations

The present findings suggest that physical fitness development among university students is a dynamic longitudinal process rather than a static state at university entry. Both baseline levels and subsequent developmental trajectories varied across physical fitness components and BMI groups, indicating substantial heterogeneity in how physical fitness evolves during university years. These findings highlight the need for long-term, component-specific, and population-targeted health promotion strategies, particularly for endurance-related fitness and students with overweight or obesity.

Although the physical fitness changes observed after 2020 coincided with the timing of the COVID-19 pandemic and related campus management measures, the interrupted time-series and cohort comparison design does not permit causal attribution and cannot distinguish the independent effects of the pandemic from underlying secular trends and other concurrent social or environmental changes. Therefore, the observed findings should be interpreted as changes observed after 2020 rather than evidence of an independent causal effect of the COVID-19 pandemic. Furthermore, because information on physical activity, sedentary behavior, sleep, and dietary behaviors was unavailable, the potential behavioral mechanisms discussed above could not be directly evaluated, and the observed associations should therefore be interpreted with caution. As the data were obtained from a single university in China, the generalizability of these findings should be interpreted with caution. Physical education curricula, campus environments, COVID-19 prevention policies, and lifestyle patterns may differ across regions, university settings, and countries, potentially resulting in different patterns of physical fitness development. Although the observed heterogeneity in physical fitness trajectories may have broader relevance, the magnitude and specific patterns of these changes require confirmation in multicenter longitudinal studies involving diverse university settings and young adult populations.

Nevertheless, this study leveraged long-term, large-scale annual physical fitness monitoring data and combined interrupted time series analysis with longitudinal trajectory analysis, providing important evidence for understanding physical fitness trends during university years and changes observed after 2020.

## 5 Conclusion

This study examined temporal trends and developmental trajectories of physical fitness among university students from 2013 to 2025. Although the post-2020 cohorts exhibited higher estimated baseline levels for several physical fitness indicators, they generally showed weaker developmental trajectories during university than the pre-2020 cohorts. Developmental patterns differed across physical fitness components and BMI groups, highlighting substantial heterogeneity in long-term physical fitness development. These findings underscore the importance of considering both baseline cohort differences and subsequent developmental trajectories when evaluating long-term changes in physical fitness and support the implementation of targeted health promotion strategies tailored to specific physical fitness components and BMI groups.

## Data Availability

The raw data supporting the conclusions of this article will be made available by the authors, without undue reservation.
