# Supplementary material for: Temporal trends and developmental trajectories of physical fitness among university students: a 13-year accelerated longitudinal study
Source: Front Public Health. 2026 Aug 19;14:1896880. doi: 10.3389/fpubh.2026.1896880 (PMC13533922; doi:10.3389/fpubh.2026.1896880)
Supplement: Supplementary file 1 [file Table_1.docx]

Supplementary Material

**Supplementary Tables**

**Table S1.** Distribution of available physical fitness assessments by enrollment cohort and academic year

| **cohort** | **Year 1 (*n*)** | **Year 2 (*n*)** | **Year 3 (*n*)** | **Year 4 (*n*)** | **Students (*n*)** | **Observations**  **(*n*)** | **Mean number of assessments** |
| --- | --- | --- | --- | --- | --- | --- | --- |
| 2012 | - | 3,284 | 3,267 | - | 3,284 | 6,551 | 1.99 |
| 2013 | 3,292 | 3,280 | 3,280 | - | 3,322 | 9,852 | 2.97 |
| 2014 | 3,226 | 3,225 | 3,216 | - | 3,226 | 9,667 | 3.00 |
| 2015 | 3,269 | 3,266 | 3,264 | 2,595 | 3,281 | 12,394 | 3.78 |
| 2016 | 3,347 | 3,345 | 2,963 | 1,496 | 3,356 | 11,151 | 3.32 |
| 2017 | 3,102 | 3,048 | 2,667 | 1,522 | 3,118 | 10,339 | 3.32 |
| 2018 | 3,365 | 3,093 | 2,770 | 1,522 | 3,738 | 10,750 | 2.88 |
| 2019 | 3,109 | 3,074 | 3,066 | 1,541 | 3,168 | 10,790 | 3.41 |
| 2020 | 3,306 | 3,321 | 3,267 | 1,476 | 3,355 | 11,370 | 3.39 |
| 2021 | 3,601 | 3,591 | 3,540 | 1,663 | 3,656 | 12,395 | 3.39 |
| 2022 | 3,978 | 4,102 | 3,909 | 1,766 | 4,162 | 13,755 | 3.3 |
| 2023 | 4,067 | 4,011 | 3,860 | - | 4,143 | 11,938 | 2.88 |
| 2024 | 4,103 | 4,034 | - | - | 4,146 | 8,137 | 1.96 |
| 2025 | 4,203 | - | - | - | 4,203 | 4,203 | 1.00 |
| Total | 45,967 | 38,372 | 35,069 | 13,581 | 50,158 | 143,292 | 2.86 |

Note: Year 1–Year 4 indicate the first to fourth annual physical fitness assessments after university enrollment. Mean assessments were calculated as the total number of observations divided by the number of students within each enrollment cohort.

**Table S2.** Distribution of the number of repeated assessments contributed by individual students across enrollment cohorts

| **cohort** | **1 assessment, n (%)** | **2 assessments, n (%)** | **3 assessments, n (%)** | **4 assessments, n (%)** |
| --- | --- | --- | --- | --- |
| 2012 | 17 (0.5) | 3,267 (99.5) | - | - |
| 2013 | 42 (1.3) | 30 (0.9) | 3,250 (97.8) | - |
| 2014 | 0 | 11 (0.3) | 3,215 (99.7) | - |
| 2015 | 15 (0.5) | 2 (0.1) | 681 (20.8) | 2,583 (78.7) |
| 2016 | 10 (0.3) | 190 (5.7) | 1,863 (55.5) | 1,293 (38.5) |
| 2017 | 37 (1.2) | 237 (7.6) | 1,548 (49.6) | 1,296 (41.6) |
| 2018 | 625 (16.7) | 374 (10.0) | 1,579 (42.2) | 1,160 (31.0) |
| 2019 | 56 (1.8) | 93 (2.9) | 1,528 (48.2) | 1,491 (47.1) |
| 2020 | 23 (0.7) | 89 (2.7) | 1,803 (53.7) | 1,440 (42.9) |
| 2021 | 42 (1.1) | 106 (2.9) | 1,891 (51.7) | 1,617 (44.2) |
| 2022 | 52 (1.2) | 285 (6.8) | 2,167 (52.1) | 1,658 (39.8) |
| 2023 | 84 (2.0) | 323 (7.8) | 3,736 (90.2) | - |
| 2024 | 155 (3.7) | 3,991 (96.3) | - | - |
| 2025 | 4,203 (100) | - | - | - |
| Total | 5,361 (10.7) | 8,998 (17.9) | 23,261 (46.4) | 12,538 (25.0) |

Note: Percentages are calculated within each enrollment cohort. One assessment indicates that a student contributed one physical fitness record during the study period, whereas four assessments indicate four repeated measurements across university years.

**Table S3.** Comparison of random-effects structures across physical fitness outcomes

| Physical Fitness Outcome | AIC (M1) | AIC (M2) | ΔAIC | LogLik (M1) | LogLik (M2) | LRT *χ*^2^ | *p*-value |
| --- | --- | --- | --- | --- | --- | --- | --- |
| Vital capacity | 333670.1 | 332567.5 | -1102.6 | -166825.1 | -166271.7 | 1106.6 | < 0.001 |
| 50-m dash | 358478.6 | 356884.4 | -1594.2 | -179229.3 | -178430.2 | 1598.2 | < 0.001 |
| Standing long jump | 339448.9 | 338589.1 | -859.9 | -169714.5 | -169282.5 | 863.9 | < 0.001 |
| Sit-and-reach | 330616.4 | 328199 | -2417.3 | -165298.2 | -164087.5 | 2421.3 | < 0.001 |
| Endurance run | 365599.3 | 362567.1 | -3032.3 | -182789.7 | -181271.5 | 3036.3 | < 0.001 |
| Upper-body muscle strength | 350441.5 | 347599.3 | -2842.2 | -175210.8 | -173787.6 | 2846.3 | < 0.001 |

M1 (random intercept model) included a random intercept for each student to account for baseline heterogeneity. M2 (random slope model) additionally included a random slope for academic grade to capture individual differences in longitudinal trajectories. AIC refers to Akaike Information Criterion, with lower values indicating better model fit. LRT refers to the likelihood ratio test comparing M1 and M2.

**Table S4.** Model fit indices for linear mixed-effects models across outcomes

| **Physical Fitness Outcome** | **Marginal R^2^** | **Conditional R^2^** | **ICC** | **RMSE** |
| --- | --- | --- | --- | --- |
| Vital capacity | 0.088 | 0.728 | 0.701 | 0.422 |
| 50-m dash | 0.018 | 0.651 | 0.645 | 0.475 |
| Standing long jump | 0.008 | 0.726 | 0.724 | 0.424 |
| Sit-and-reach | 0.072 | 0.748 | 0.729 | 0.393 |
| Endurance run | 0.061 | 0.624 | 0.6 | 0.502 |
| Upper-body muscle strength | 0.05 | 0.675 | 0.658 | 0.449 |

Marginal R^2^ represents the proportion of variance explained by fixed effects (age, assessment year, academic grade, and cohort).

Conditional R^2^ represents the proportion of variance explained by both fixed and random effects.

ICC (intraclass correlation coefficient) represents the proportion of total variance attributable to between-subject differences.

RMSE (root mean square error) represents the average prediction error in Z-score units.

**Table S5.** Baseline characteristics of students with complete and incomplete follow-up (2015–2022)

| **Variables** | **Complete follow-up** | **Incomplete follow-up** | **SMD** |
| --- | --- | --- | --- |
| **Overall** |  |  |  |
| Students, *n* | 12538 | 14539 |  |
| Age (years), mean (SD) | 17.88 (0.89) | 18.01 (1.05) | 0.137 |
| Female sex, n (%) | 5,995 (47.8) | 8,040 (55.3) | 0.15 |
| BMI (kg/m^2^), mean (SD) | 21.69 (3.24) | 21.73 (3.42) | 0.012 |
| **BMI category, n (%)** |  |  | 0.044 |
| Underweight | 1,604 (12.8) | 1,961 (13.5) |  |
| Normal weight | 8,493 (67.7) | 9,631 (66.2) |  |
| Overweight | 1,846 (14.7) | 2,141 (14.7) |  |
| Obesity | 595 (4.7) | 806 (5.5) |  |
| Total physical fitness score, mean (SD) | 72.72 (8.38) | 72.61 (8.60) | 0.012 |
| **Male students** |  |  |  |
| 1000-m run (m/s) | 4.08 (0.47) | 4.04 (0.48) | 0.079 |
| 50-m dash (m/s) | 6.64 (0.46) | 6.63 (0.47) | 0.031 |
| Standing long jump (cm) | 226.63 (20.87) | 225.72 (21.71) | 0.043 |
| Vital capacity (mL) | 4120.50 (651.08) | 4108.15 (678.52) | 0.019 |
| Sit-and-reach (cm) | 16.11 (7.16) | 16.33 (6.84) | 0.032 |
| Pull-ups (repetitions) | 4.43 (4.54) | 4.53 (4.59) | 0.02 |
| **Female students** |  |  |  |
| 800-m run (m/s) | 3.44 (0.36) | 3.40 (0.37) | 0.113 |
| 50-m dash (m/s) | 5.45 (0.38) | 5.42 (0.38) | 0.071 |
| Standing long jump (cm) | 172.65 (16.65) | 170.91 (16.88) | 0.103 |
| Vital capacity (mL) | 2803.55 (479.78) | 2810.81 (507.98) | 0.015 |
| Sit-and-reach (cm) | 20.06 (5.98) | 19.83 (5.87) | 0.038 |
| Sit-ups (repetitions) | 32.68 (9.31) | 32.32 (8.82) | 0.039 |

Note: SD, standard deviation; SMD, standardized mean difference. Complete follow-up was defined as four physical fitness assessments, whereas incomplete follow-up was defined as fewer than four assessments among students enrolled between 2015 and 2022 who were eligible to complete four annual assessments by the end of the study period. SMD values of 0.2, 0.5, and 0.8 were considered indicative of small, moderate, and large differences, respectively.

**Table S6.** Potential temporal change points identified by exploratory segmented regression analysis

| Fitness indicator | Gender | Estimated change point (year) |
| --- | --- | --- |
| Endurance run | Male | 2023 |
|  | Female | 2022 |
| Standing long jump | Male | Not identified |
|  | Female | Not identified |
| BMI | Male | 2020 |
|  | Female | 2020 |
| Vital capacity | Male | Not identified |
|  | Female | Not identified |
| 50-m dash | Male | Not identified |
|  | Female | Not identified |
| Sit-and-reach | Male | 2019 |
|  | Female | 2021 |
| Upper-body muscle strength | Male | Not identified |
|  | Female | Not identified |

**Note:** Change points were identified using exploratory segmented regression analysis. An identified change point represents the year at which the segmented regression model detected a temporal change. "Not identified" indicates that no change point was identified for the corresponding fitness indicator.

**Table S7.** Fixed-effect estimates from the final linear mixed-effects models examining interactions among BMI category, enrollment cohort, and academic grade for physical fitness trajectories.

| **Variables** | **Endurance run** | **50-m dash** | **Standing long jump** | **Upper-body** muscle strength | **Vital capacity** | **Sit-and-reach** |
| --- | --- | --- | --- | --- | --- | --- |
| Intercept | **−0.263** (0.015) | **−0.380** (0.015) | **−0.278** (0.014) | **−0.690** (0.014) | **−0.889** (0.014) | **−0.636** (0.013) |
| Age | **−0.027** (0.004) | **−0.033** (0.004) | **−0.027** (0.004) | **−0.013** (0.004) | 0.006 (0.004) | −0.007 (0.004) |
| Assessment year | **−0.065** (0.002) | **−0.050** (0.002) | **−0.018** (0.002) | **0.016** (0.002) | **0.064** (0.002) | **0.069** (0.002) |
| **Grade effects** |  |  |  |  |  |  |
| Grade | **0.604** (0.013) | **0.522** (0.013) | **0.276** (0.011) | **0.301** (0.012) | **0.308** (0.011) | **0.102** (0.011) |
| Grade^2^ | **−0.106** (0.003) | **−0.080** (0.003) | **−0.033** (0.002) | **−0.022** (0.002) | **−0.041** (0.002) | **0.004** (0.002) |
| **Cohort effect** |  |  |  |  |  |  |
| Post-2020 cohort | **0.881** (0.025) | **0.737** (0.025) | 0**.139** (0.024) | **0.363** (0.024) | **0.277** (0.023) | **−0.116** (0.023) |
| **BMI Main Effects** |  |  |  |  |  |  |
| Underweight | −0.034 (0.019) | **−0.041** (0.019) | **0.042** (0.017) | −0.023 (0.018) | **−0.247** (0.017) | **−0.199** (0.017) |
| Overweight | **−0.277** (0.022) | **−0.214** (0.022) | **−0.187** (0.020) | **−0.155** (0.021) | **0.309** (0.020) | −0.009 (0.020) |
| Obese | **−0.648** (0.037) | **−0.568** (0.037) | **−0.496** (0.034) | **−0.225** (0.036) | **0.385** (0.034) | **−0.075** (0.034) |
| **Grade × Cohort interactions** |  |  |  |  |  |  |
| Grade × Post-2020 cohort | **−0.403** (0.019) | **−0.353** (0.018) | 0.032 (0.016) | 0.021 (0.018) | **−0.084** (0.016) | **0.217** (0.016) |
| Grade^2^ × Post-2020 cohort | **0.026** (0.004) | **0.060** (0.004) | **−0.022** (0.003) | **−0.028** (0.004) | −0.001 (0.003) | **−0.068** (0.003) |
| **Grade × BMI Interactions** |  |  |  |  |  |  |
| Grade × Underweight | **0.028** (0.008) | **0.022** (0.008) | −0.004 (0.007) | 0.011 (0.008) | 0.008 (0.007) | **0.038** (0.007) |
| Grade × Overweight | −0.005 (0.009) | **−0.022** (0.008) | **−0.019** (0.007) | **−0.051** (0.008) | **−0.061** (0.007) | **−0.039** (0.007) |
| Grade × Obese | **−0.034** (0.015) | −0.026 (0.014) | −0.001 (0.012) | **−0.103** (0.014) | **−0.085** (0.012) | **−0.082** (0.012) |
| **Post-2020 Cohort × BMI Interactions** |  |  |  |  |  |  |
| Post-2020 × Underweight | **0.082** (0.032) | **0.089** (0.032) | 0.038 (0.029) | **0.164** (0.031) | 0.006 (0.029) | **0.091** (0.029) |
| Post-2020 × Overweight | **−0.102** (0.030) | −0.047 (0.030) | **−0.064** (0.027) | **−0.225** (0.029) | **−0.165** (0.027) | **−0.108** (0.027) |
| Post-2020 × Obese | **−0.321** (0.049) | −0.041 (0.049) | −0.013 (0.044) | **−0.384** (0.047) | **−0.191** (0.044) | **−0.238** (0.044) |
| **Grade × Post-2020 Cohort × BMI** |  |  |  |  |  |  |
| Grade × Post-2020 × Underweight | **−0.076** (0.014) | **−0.045** (0.014) | −0.004 (0.012) | **−0.035** (0.013) | −0.023 (0.012) | **−0.033** (0.012) |
| Grade × Post-2020 × Overweight | **0.036** (0.013) | 0.022 (0.012) | 0.020 (0.011) | **0.050** (0.012) | **0.032** (0.011) | 0.012 (0.011) |
| Grade × Post-2020 × Obese | **0.111** (0.021) | 0.031 (0.020) | −0.010 (0.017) | **0.089** (0.019) | **0.037** (0.017) | 0.026 (0.017) |

Note：Values are presented as fixed-effect estimates *β* (SE). Normal-weight participants and pre-2020 cohorts were used as reference groups.

**Supplementary Figures**

**Figure S1**

Estimated longitudinal trajectories of endurance run performance across university grades stratified by BMI categories

Solid lines represent estimated marginal means, and shaded areas indicate 95% confidence intervals. Panels indicate baseline BMI categories. G1–G4 denote academic grades 1–4. All outcomes were standardized as sex-specific Z-scores, with higher values indicating better physical fitness performance.

**Figure S2**

Estimated longitudinal trajectories of upper body muscle strength across university grades stratified by BMI categories.

Solid lines represent estimated marginal means, and shaded areas indicate 95% confidence intervals. Blue and red lines represent the pre-2020 cohort and post-2020 cohort, respectively. G1–G4 denote academic grades 1–4, and panel headings indicate baseline BMI categories. All outcomes were standardized as sex-specific Z-scores, with higher values indicating better physical fitness performance.
